# Neonate, Infant, and Child Mortality by Cause in Provinces of Iran: An Analysis for the Global Burden of Disease Study 2019

**DOI:** 10.34172/aim.2022.80

**Published:** 2022-08-01

**Authors:** Sadaf G. Sepanlou, Hossein Rezaei Aliabadi, Mohsen Naghavi, Reza Malekzadeh, GBD 2019 Iran Child Collaborators

**Affiliations:** ^1^Digestive Disease Research Institute, Tehran University of Medical Sciences, Tehran, Iran; ^2^Bam University of Medical Sciences, Bam, Iran; ^3^Institute for Health Metrics and Evaluation, School of Medicine, University of Washington, Seattle, USA

**Keywords:** Child mortality, Global Burden of Disease, Infant mortality, Iran, Sustainable development

## Abstract

**Background:**

Since 1990, neonatal, infant, and child mortality has substantially decreased in Iran. However, estimates for mortality by cause at subnational scale are not available.

**Methods:**

This study is part of the Global Burden of Diseases, Injuries, and Risk Factors study (GBD) 2019. Here we report the number and rates of neonate, infant, and child deaths by cause across provinces of Iran from 1990 to 2019.

**Results:**

Between 1990 and 2019, the neonatal mortality rate per 1000 live births decreased from 31.8 (95% UI: 28.1–35.5) to 6.8 (6.1–7.4). The child mortality rates decreased from 71.2 (63.6–79.1) to 11.1 (10.2–12.0) per 1000 live births. Mortality rates among neonates per 1000 live births ranged from 3.1 (2.6–3.7) to 10.0 (9.2–10.8) across provinces in 2019. Child mortality rate per 1000 live births ranged from 5.5 (4.6–6.5) to 17.9 (16.4–19.4) across provinces in 2019. Neonatal disorders, congenital birth defects, and lower respiratory infections were the three main causes of mortality in almost all provinces of Iran. The majority of neonatal disorders were due to neonatal preterm birth and neonatal asphyxia, trauma, and infections. The trends of mortality across provinces from 1990 to 2019 were converging and decreased along with increase in sociodemographic index (SDI).

**Conclusions:**

All provinces achieved the Sustainable Development Goal 3.2 of neonatal mortality less than 12 and child mortality less than 25 per 1000 live births. However, disparities still exist across provinces, specifically in low-SDI provinces.

## Introduction

 Infant and child mortality are among the main indicators of health and socioeconomic development in nations. The latest results of the Global Burden of Diseases, Injuries, and Risk Factors Study (GBD 2019) demonstrated that in 2019, the total global number of deaths among children under 5 years old was slightly over 5 million, showing a 58% decrease from 12 million in 1990.^1,2^ Quite similar estimates have been reported by the United Nations International Children’s Emergency Fund (UNICEF), reporting 5.2 million child mortality in 2019.^3^ Despite the success during the past three decades at global level, neonatal, infant, and child mortality are still challenging in many countries, including those located in north Africa and the Middle East.^4^ Out of the global burden of 5 million deaths among children under 5 years in 2019, 2.7 million (more than half) occurred in countries with low sociodemographic index (SDI), 1.5 million occurred in countries with low-middle SDI, 685 thousand were observed in middle SDI countries, and less than 200 thousand occurred among countries with high-middle SDI and high SDI combined.^1,2,5,6^ UNICEF reports that out of 5.2 million deaths among children at global level in 2019, more than half occurred in sub-Saharan Africa.^3^ With the release of Sustainable Development Goals (SDGs) in 2015, targets set for optimal neonate and child mortality rates were 12 and 25 deaths per 1000 live births respectively.^7,8^ But it seems that many low and middle-income countries are unlikely to achieve these targets by 2030.^9,10^

 In Iran with a high-middle SDI, we also witnessed a significant decrease in neonate, infant, and child mortality during the past three decades. The reports of UNICEF in 2019 showed over 70% decrease in neonate, infant, and child mortality rate at national level in Iran since 1990.^3,11^ This report showed that infant mortality rate per 1000 live births in Iran decreased from 43.7 in 1990 to 12.0 in 2019. Respective figures for under-5 mortality rate were 56.1 and 13.9 per 1000 live births. There are a number of other studies that have estimated neonate or child mortality at national or subnational levels in Iran.^12-18^ However, they had certain limitations. They either did not use robust methods to overcome the incompleteness of the death registration system in Iran, were cross-sectional, did not report both neonate, infant, and child mortality rate by cause, or did not report estimates at subnational level in Iran. A recent Iranian study showed that under 5 mortality rate (U5MR) per 1000 live births decreased from 63.6 to 19.4 from 1990 to 2015.^19^ It was a comprehensive study in Iran that reported national and subnational estimates for under-5 mortality from 1990 to 2015,^20^ using summary birth history data from different sources and using maternal age cohort and maternal age period methods to estimate the trends in child mortality rates. Yet, it didn’t estimate neonatal and infant mortality and didn’t report mortality by cause. In previous iterations of GBD, child mortality has been estimated at national level in Iran using several data sources and robust methods to overcome data scarcity and incompleteness.^21-23^ To the best of our knowledge, the current study is the first that reports all of the neonatal, infant, and child mortality rates by cause at national and subnational level in Iran from 1990 to 2019.

## Materials and Methods

 This study is part of the GBD 2019 study, which was a systematic effort to estimate the levels, trends, and causes of mortality by sex, age, year (1990 to 2019), and location. In this article we report the estimates for neonate (0–27 days), infant (under 1 year), and child (under 5 year) mortality by cause at national level in Iran along with sub-national estimates for 31 provinces.

###  All-Cause Mortality

 We used multiple data types to estimate all-cause mortality rate in Iran, including data from vital registration system (32 data sources), and surveys and censuses, from which we extracted household death recall data (9 data sources), summary birth histories (5 data sources), and complete birth histories (one data source).^10^ First, the ratio of children ever surviving to children ever born by age of mother was converted to an estimate of U5MR. Second, a multi-stage process was used to include a non-linear mixed effects model, source bias correction, spatiotemporal smoothing, and Gaussian process regression to synthesize U5MR data into both sex U5MR estimates for every location-year in Iran. Complete time series of lag-distributed income, maternal education (mean years of education among women of reproductive age, 15–49 years), and childhood HIV crude were incorporated into the mixed effects model. Third, analogous multi-stage location-year specific models were used to estimate the sex-ratio of under-5 mortality and age- and sex specific probability of death for neonates and infants. Fourth, death counts were calculated by simulating exposure to the estimated age-specific mortality probabilities for weekly birth cohorts from GBD 2019 live birth estimates. Fifth, death counts were divided by corresponding age-specific mortality rates (deaths per person-year) which is the version of the mortality metric presented in most GBD publications and visualization tools. Finally, fatal discontinuities, defined as sharp change in mortality by year resulting from unexpected events such as natural disasters and conflict, were estimated separately and appended to generate final all-cause mortality estimates.^10^

###  Cause-Specific Mortality

 For calculating cause specific mortality, cause of death data from above mentioned data sources were in the first step mapped to the GBD cause list using various revisions of the International Classification of Diseases and Injuries.^1^ Second, data was corrected for misclassification to non-specific codes, impossible causes, or clinical syndromes by redistributing these so-called “garbage codes” to the most likely underlying causes of death by age, sex, location, and year using a number of previously described statistical approaches.^1,10^ Third, a non-zero floor was calculated defined as + /- 2 median absolute deviations for each age-sex-cause combination to reduce downward bias that could be introduced by redistribution for comparatively rare causes in locations with good data quality. Fourth, a Bayesian noise-reduction algorithm was applied based on a Poisson count model to account for stochastic variation in data and reduce the upward bias that could be introduced by zero counts.^1,10^

 After completion of data processing, a complete time series of mortality estimates was generated for each cause. Cause of Death Ensemble modeling (CODEm) was the most commonly-used modeling platform. CODEm uses a train-test-test approach to evaluate multiple submodels, each of which contain different combinations of candidate covariates, to systematically maximize out of sample predictive validity and weight them accordingly in generating a final ensemble model.^24^ Cause-specific mortality estimates were then scaled to match all-cause mortality estimates for each age group, location, sex, and year in a process called CoDCorrect.^1^ Similar to the all-cause mortality analysis above, cause-specific deaths due to fatal discontinuities (e.g., conflict and terrorism, major transportation accidents, natural disasters, epidemics, other forms of disaster such as large fires or the collapse of large buildings) were estimated separately using vital registration and alternative databases and appended to final results. Cause-specific mortality rates were converted into probabilities by multiplying all-cause probability of death by cause fraction.

###  Disparity in Mortality

 We used the SDI to determine the relationship between the development level of a province and infant and child mortality. SDI is a composite indicator of a province’s lag-distributed income per capita, average years of schooling among individuals over 15 years old, and the fertility rate in females under the age of 25 years.^2^

 The methods we used for propagating uncertainty are the same as those used in previous GBD iterations.^1,25^ All all-cause and cause-specific mortality estimation components are based on 1000 draws, or simulations, by age, sex, location, and year. Point estimates were derived from the mean of the draws, and 95% uncertainty intervals (UIs) were calculated as the 2.5^th^ and 97.5^th^ percentiles of the draws.

## Results

###  National Estimates

 In 1990, a total of 55 513 (95% UI: 46 255–65 088) deaths occurred among neonates in Iran, which decreased to 9145 (7444–11 136) deaths in 2019. During the same time interval, the neonatal mortality rate declined from 31.8 (28.1, 35.5) per 1000 live births to 6.8 (6.1, 7.4) per 1000 live births (Figure 1).

**Figure 1:**
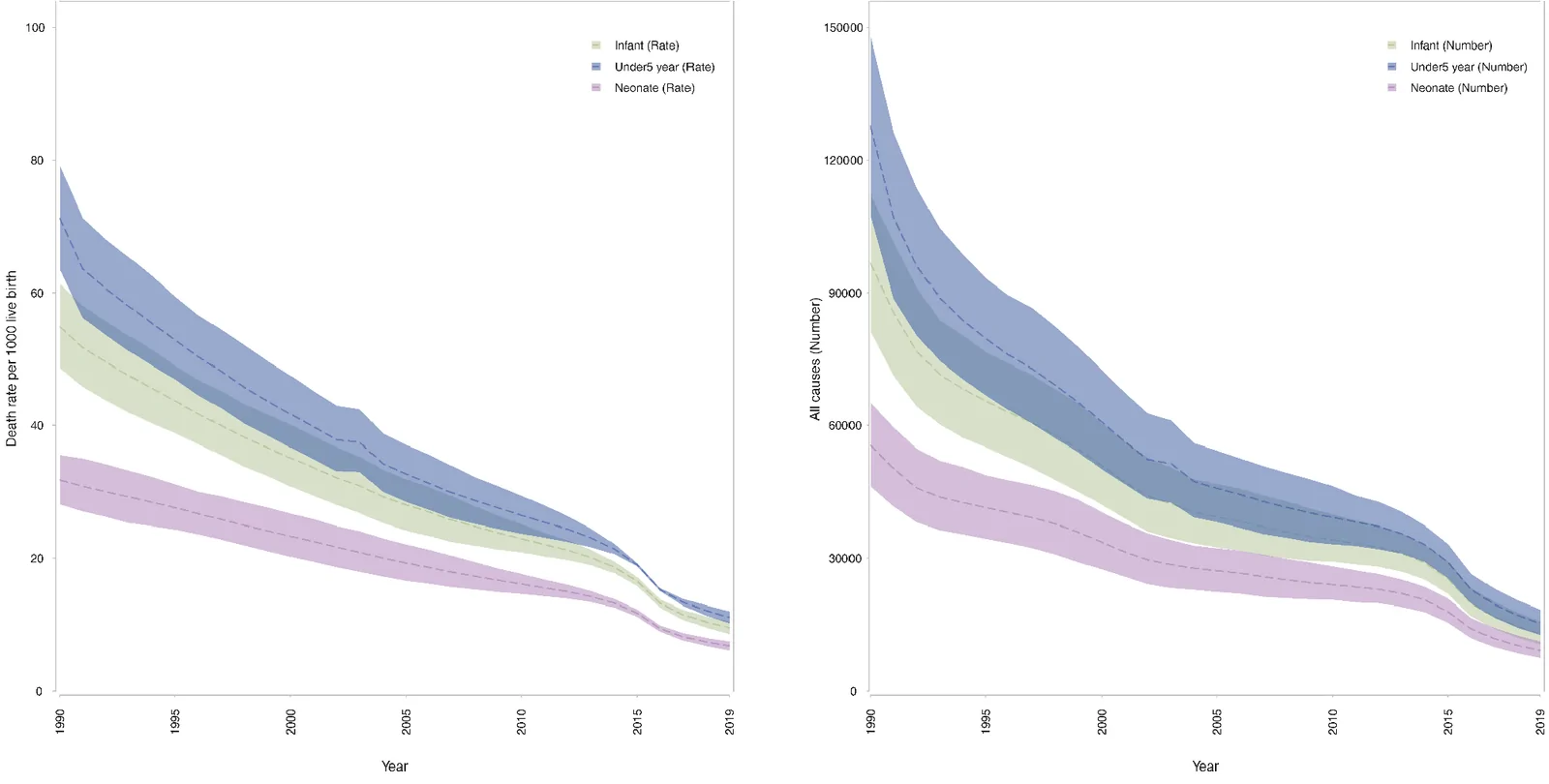
The Trend in Neonate, Infant, and Child Mortality Counts and Rates at National Level from 1990 to 2019 in Iran

 In 1990, a total of 96 630 (95% UI: 81 362–112 155) deaths occurred among infants under 1 year in Iran, which decreased to 12 937 (10 548–15 718) deaths in 2019. The rate of infant mortality decreased as well from 54.9 (48.7–61.3) per 1000 live births in 1990 to 9.5 (8.6–10.5) per 1000 live births in 2019 (Figure 1).

 Respective figures for U5MR show similar decline. In 1990 a total of 127 608 (107 686–147 945) deaths occurred among children under 5 years in Iran, which declined to 15 231 (12 690–18 363) deaths in 2019. Respective figures for U5MRs were 71.2 (63.6–79.1) in 1990 and 11.1 (10.2–12.0) per 1000 live births in 2019 (Figure 1). The majority of deaths among children under 5 years were due to neonatal deaths (43.5% in 1990 and 60.0% in 2019) and under 1 year infant deaths (75.7% in 1990 and 84.9% in 2019).

Figure 2 demonstrates that neonatal, infant, and child mortality rates were higher in males than females in all years from 1990 to 2019 across Iran. The same pattern was observed in all provinces.

**Figure 2:**
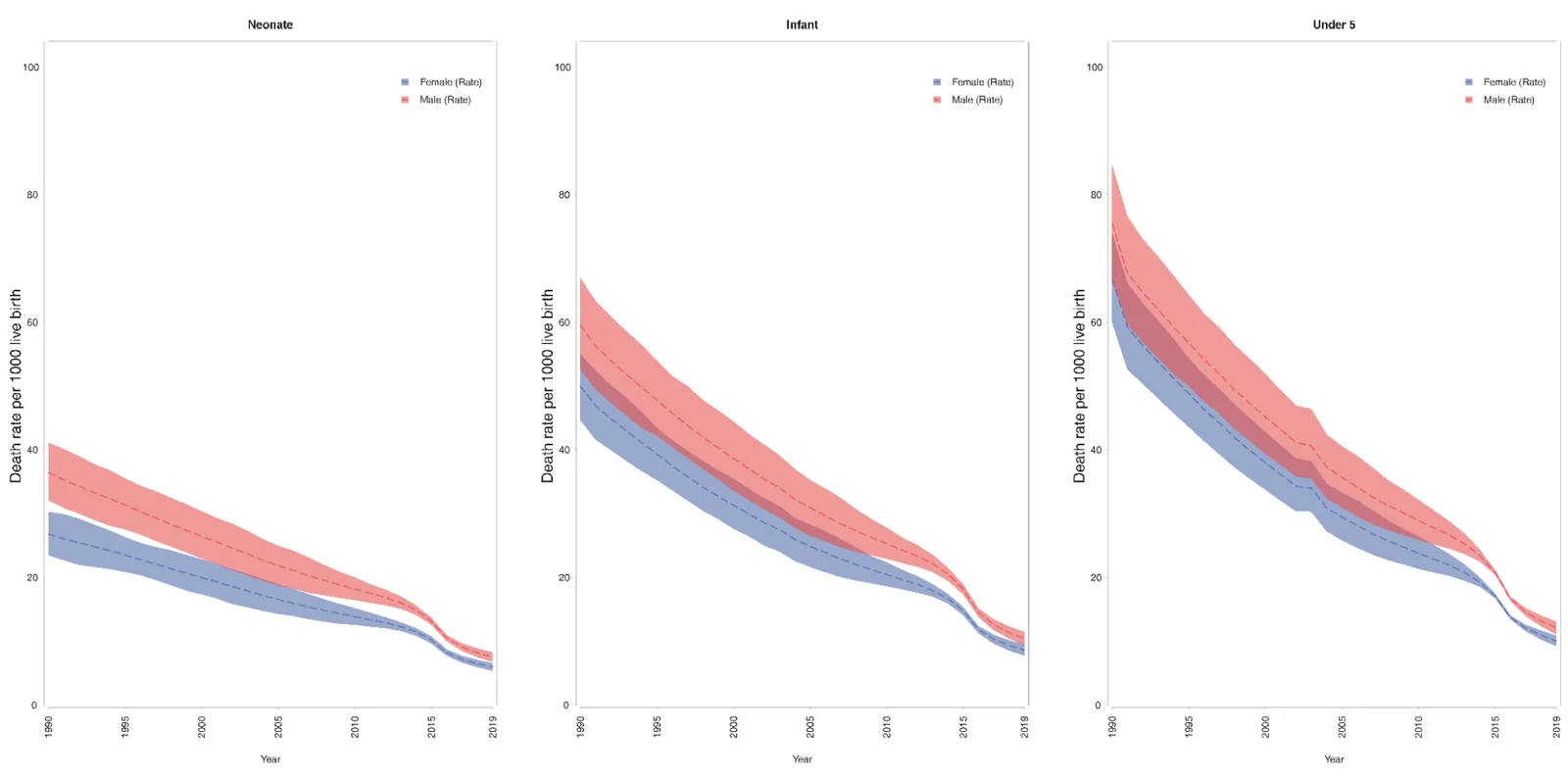
Neonatal, Infant, and Child Mortality Rates by Sex in Iran from 1990 to 2019

###  Provincial Estimates

 In 2019, the mortality rate among neonates ranged from 3.1 (2.6–3.7) per 1000 live births in Gilan to 10.0 (9.2–10.8) per 1000 live births in Sistan and Baluchistan (Figure 3A, Table 1). All provinces of Iran achieved the SDG 3.2 target of neonatal mortality less than 12 deaths per 1000 live births in 2019. The highest decline in neonatal mortality rate during the past three decades was observed in Gilan (-87.8%) and the lowest occurred in Kerman (-71.5%) (Table 1).

**Figure 3:**
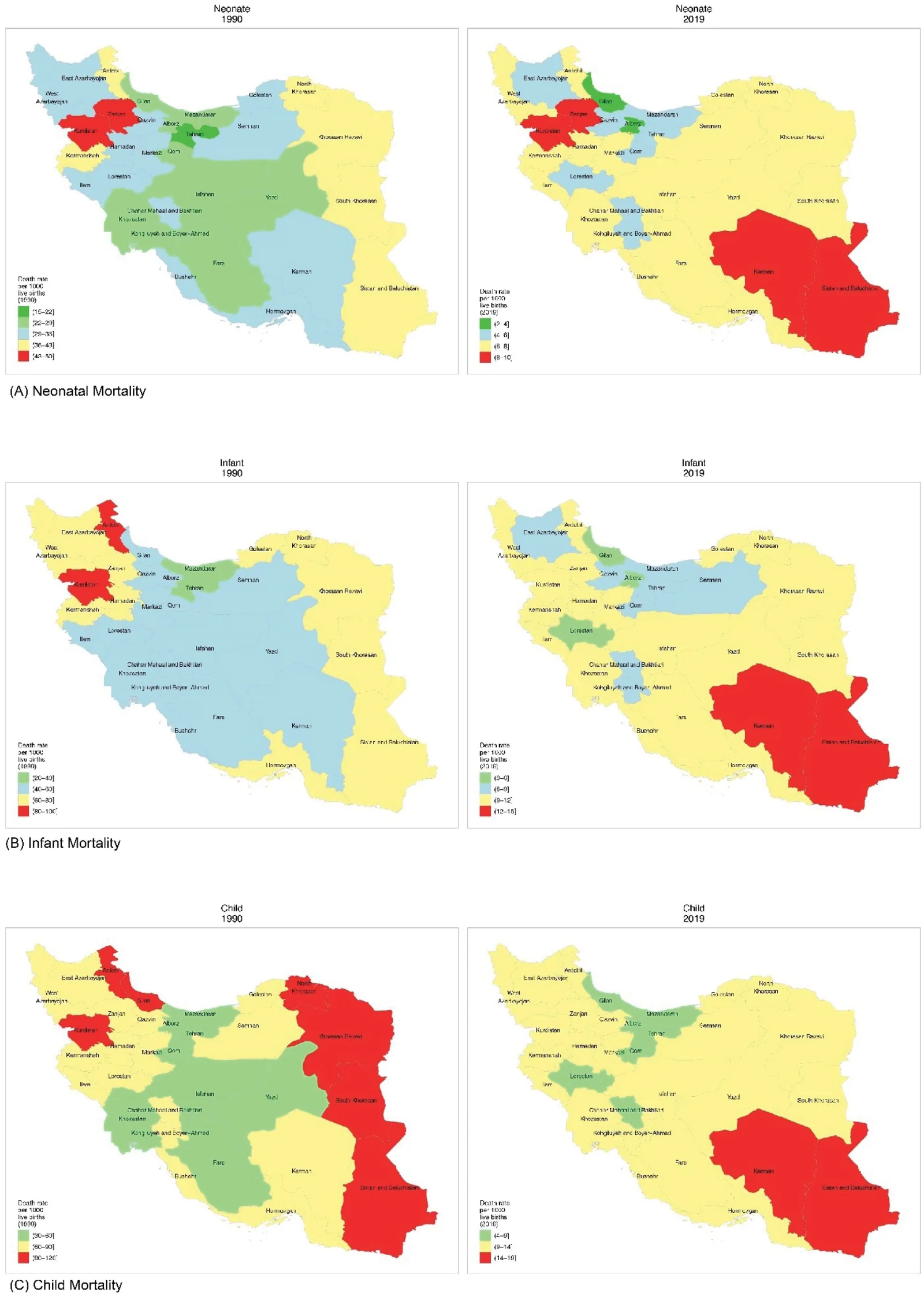
Neonatal, Infant, and Child Mortality Rates Across Provinces of Iran in 1990 and 2019. (**A)** Neonates; (**B)** Infants; (**C)** Children

**Table 1:** Neonatal Deaths and Neonate Mortality Per 1000 Live Births in 1990 and 2019 and the Percent Change in Mortality Rate from 1990 to 2019

**Location**	**Count 1990**	**Rate 1990**	**Count 2019**	**Rate 2019**	**Percent Change**
Iran	55513 (46255, 65088)	31.8 (28.1, 35.5)	9145 (7444, 11136)	6.8 (6.1, 7.4)	-78.6
Alborz	1344 (1101, 1620)	24.1 (20.8, 27.7)	231 (184, 286)	3.9 (3.6, 4.3)	-83.7
Ardebil	1507 (1241, 1797)	40 (34.6, 45.8)	152 (124, 187)	7.2 (6.6, 7.8)	-82
Bushehr	822 (657, 1025)	30.6 (25.4, 36.9)	157 (122, 202)	7 (6, 8.1)	-77.1
Chahar Mahaal and Bakhtiari	758 (628, 908)	29.1 (25.1, 33.3)	81 (64, 103)	4.3 (3.7, 5)	-85
East Azarbayejan	3389 (2822, 4027)	32.8 (28.5, 37.5)	339 (275, 416)	5.3 (4.9, 5.8)	-83.8
Fars	2877 (2375, 3454)	25.8 (22.4, 29.6)	537 (433, 661)	6.9 (6.3, 7.4)	-73.4
Gilan	1609 (1225, 2048)	25.8 (20.5, 32)	97 (75, 124)	3.1 (2.6, 3.7)	-87.8
Golestan	1549 (1286, 1844)	32.4 (28.2, 37.1)	254 (207, 312)	7 (6.4, 7.5)	-78.5
Hamadan	1850 (1526, 2218)	35.2 (30.4, 40.3)	199 (161, 245)	7.5 (6.9, 8.1)	-78.7
Hormozgan	1206 (1003, 1436)	33.3 (28.6, 38.4)	280 (228, 344)	6.9 (6.4, 7.5)	-79.1
Ilam	516 (428, 617)	31.4 (27.2, 36.2)	64 (49, 82)	7 (5.8, 8.4)	-77.6
Isfahan	2565 (2058, 3178)	26 (21.8, 31)	409 (338, 496)	6.3 (5.8, 6.8)	-75.7
Kerman	1838 (1501, 2218)	29.5 (25.5, 34)	528 (433, 645)	8.4 (7.7, 9.1)	-71.5
Kermanshah	2068 (1716, 2482)	37.7 (32.8, 43.2)	249 (201, 306)	7.7 (7, 8.3)	-79.7
Khorasan-e-Razavi	6258 (5215, 7433)	36.9 (32, 42.3)	934 (752, 1147)	6.9 (6.4, 7.5)	-81.2
Khuzestan	3175 (2645, 3752)	27.3 (24.1, 30.9)	688 (559, 840)	6.8 (6.3, 7.4)	-75
Kohgiluyeh and Boyer-Ahmad	584 (487, 690)	28.7 (24.8, 33)	87 (69, 111)	5.5 (4.7, 6.4)	-80.9
Kurdistan	1970 (1652, 2321)	48 (42.3, 54)	238 (193, 291)	8.4 (7.7, 9.1)	-82.4
Lorestan	1713 (1442, 1993)	30 (26.2, 34.1)	128 (101, 164)	4 (3.5, 4.7)	-86.5
Markazi	1015 (833, 1208)	31 (27, 35.3)	123 (97, 155)	6.2 (5.2, 7.2)	-80.1
Mazandaran	1759 (1436, 2124)	23.2 (19.7, 26.9)	200 (164, 241)	4.5 (4.1, 4.9)	-80.4
North Khorasan	1057 (879, 1253)	38.6 (33.5, 44.4)	118 (93, 150)	7.1 (6, 8.2)	-81.7
Qazvin	866 (716, 1023)	32 (27.9, 36.3)	118 (93, 150)	5.6 (4.8, 6.5)	-82.5
Qom	551 (445, 662)	24.2 (20.4, 28)	108 (85, 138)	4.5 (3.9, 5.2)	-81.3
Semnan	394 (324, 469)	30.9 (26.9, 35.2)	71 (55, 91)	6.1 (5.1, 7.3)	-80.2
Sistan and Baluchistan	2401 (2042, 2801)	38.6 (33.6, 43.9)	920 (750, 1116)	10 (9.2, 10.8)	-74.1
South Khorasan	1014 (831, 1205)	39.7 (34.6, 45.1)	128 (101, 163)	7.6 (6.5, 8.8)	-80.7
Tehran	4235 (3167, 5459)	17.3 (13.4, 22)	932 (760, 1136)	4.5 (4.1, 4.8)	-74.2
West Azarbayejan	2789 (2316, 3322)	35.5 (30.8, 40.6)	472 (383, 578)	7 (6.4, 7.6)	-80.3
Yazd	572 (470, 681)	26.8 (22.9, 30.9)	158 (125, 199)	7.5 (6.4, 8.7)	-71.9
Zanjan	1265 (1068, 1479)	46.2 (40.6, 52.7)	143 (113, 181)	8.1 (6.9, 9.4)	-82.5

 The mortality rate among infants under 1 year ranged from 4.7 (3.9–5.6) per 1000 live births in Gilan to 12.8 (11.7–13.9) in Kerman and 14.9 (13.7–16.2) per 1000 live births in Sistan and Baluchistan in 2019 (Figure 3B, Table 2). The highest decline in infant mortality rate between 1990 and 2019 was observed in Gilan (-90.6%) and the lowest decline was estimated for the capital province of Tehran (-74.6%) (Table 2).

**Table 2:** Infant Deaths and Infant Mortality Per 1000 Live Births in 1990 and 2019 and the Percent Change in Mortality Rate from 1990 to 2019

**Location**	**Count 1990**	**Rate 1990**	**Count 2019**	**Rate 2019**	**Percent Change**
Iran	96630 (81362, 112155)	54.9 (48.7, 61.3)	12937 (10548, 15718)	9.5 (8.6, 10.5)	-82.7
Alborz	2127 (1746, 2546)	40.2 (34.6, 46.1)	329 (262, 407)	6 (5.4, 6.5)	-85.2
Ardebil	2833 (2363, 3337)	80.2 (69.4, 91.5)	219 (178, 268)	11 (10.1, 11.8)	-86.3
Bushehr	1388 (1097, 1736)	54.5 (45.2, 65.6)	225 (174, 289)	10.7 (9.1, 12.3)	-80.4
Chahar Mahaal and Bakhtiari	1264 (1044, 1504)	51.1 (44.2, 58.6)	116 (91, 147)	6.6 (5.6, 7.6)	-87.2
East Azarbayejan	6059 (5051, 7215)	61.8 (53.7, 70.7)	486 (396, 597)	8.1 (7.4, 8.7)	-87
Fars	4754 (3933, 5638)	44.7 (38.8, 51.1)	774 (625, 952)	10.5 (9.6, 11.3)	-76.6
Gilan	3037 (2429, 3747)	50.3 (41.6, 60.3)	137 (106, 176)	4.7 (3.9, 5.6)	-90.6
Golestan	2747 (2294, 3238)	60.9 (53.1, 69.7)	365 (298, 447)	10.6 (9.7, 11.4)	-82.7
Hamadan	3363 (2777, 4019)	67.7 (58.6, 77.4)	287 (233, 351)	11.4 (10.4, 12.3)	-83.2
Hormozgan	2173 (1804, 2607)	64 (55.1, 73.7)	402 (328, 493)	10.6 (9.7, 11.5)	-83.5
Ilam	900 (752, 1062)	58.2 (50.6, 66.9)	92 (71, 118)	10.7 (8.9, 12.7)	-81.7
Isfahan	4108 (3271, 5066)	43.4 (36.4, 51.8)	587 (484, 709)	9.6 (8.8, 10.3)	-78
Kerman	3155 (2580, 3741)	53.4 (46.2, 61.3)	760 (625, 929)	12.8 (11.7, 13.9)	-76
Kermanshah	3783 (3160, 4485)	72.9 (63.6, 83.6)	360 (291, 441)	11.7 (10.7, 12.7)	-84
Khorasan-e-Razavi	11579 (9714, 13720)	72.4 (63, 82.8)	1340 (1083, 1645)	10.5 (9.7, 11.4)	-85.4
Khuzestan	5132 (4332, 6017)	46.7 (41.4, 52.6)	962 (783, 1174)	10.1 (9.2, 10.9)	-78.4
Kohgiluyeh and Boyer-Ahmad	983 (823, 1154)	51.5 (45.3, 58)	118 (93, 151)	7.9 (6.7, 9.1)	-84.7
Kurdistan	3657 (3099, 4232)	95.3 (85.1, 106)	315 (256, 384)	11.7 (10.7, 12.7)	-87.7
Lorestan	2793 (2362, 3236)	51.7 (45.5, 58.4)	175 (138, 223)	5.8 (5, 6.7)	-88.7
Markazi	1837 (1533, 2167)	59.1 (52.5, 66.4)	181 (142, 230)	9.6 (8.2, 11.2)	-83.7
Mazandaran	2566 (2110, 3073)	35.1 (30, 40.8)	266 (218, 322)	6.3 (5.8, 6.9)	-81.9
North Khorasan	1946 (1617, 2315)	76.3 (66.4, 87.7)	169 (133, 216)	10.7 (9.2, 12.4)	-86
Qazvin	1513 (1272, 1768)	59.2 (52.4, 66.2)	166 (131, 210)	8.3 (7.1, 9.6)	-86
Qom	938 (769, 1120)	43.2 (36.6, 49.9)	160 (126, 204)	7.1 (6.1, 8.2)	-83.5
Semnan	640 (533, 752)	52.6 (46, 59.6)	95 (74, 122)	8.7 (7.2, 10.3)	-83.4
Sistan and Baluchistan	4528 (3913, 5246)	78.5 (69.2, 88.5)	1302 (1065, 1580)	14.9 (13.7, 16.2)	-80.9
South Khorasan	1920 (1605, 2259)	79.8 (70.2, 90.5)	182 (144, 231)	11.5 (9.8, 13.3)	-85.6
Tehran	6080 (4705, 7652)	25.9 (20.4, 32.1)	1295 (1058, 1578)	6.6 (6, 7.1)	-74.6
West Azarbayejan	5065 (4201, 6055)	68.5 (59.5, 78.1)	677 (549, 827)	10.6 (9.8, 11.5)	-84.5
Yazd	871 (730, 1027)	42.7 (37.3, 48.8)	212 (168, 267)	10.7 (9.1, 12.3)	-75
Zanjan	2891 (2603, 3206)	69.2 (61, 78.1)	182 (143, 229)	10.7 (9.1, 12.4)	-84.5

 Similarly, in 2019, the U5MR ranged from 5.5 (4.6–6.5) per 1000 live births in Gilan to 14.8 (13.6 – 16.0) in Kerman and 17.9 (16.4 –19.4) per 1000 live births in Sistan and Baluchistan (Figure 3C, Table 3). In 2019, all provinces of Iran achieved the SDG 3.2 target of under 5 mortality less than 25 deaths per 1000 live births. The highest decline in U5MR since 1990 was observed in Gilan (-94.5%) and the lowest (-76.4%) in Tehran and Yazd (Table 3).

**Table 3:** Child Deaths and Child Mortality Per 1000 Live Births in 1990 and 2019 and the Percent Change in Mortality Rate from 1990 to 2019

**Location**	**Count 1990**	**Rate 1990**	**Count 2019**	**Rate 2019**	**Percent Change**
Iran	127608 (107686, 147945)	71.2 (63.6, 79.1)	15231 (12690, 18363)	11.1 (10.2, 12.0)	-84.4
Alborz	2610 (2156, 3139)	49.5 (42.7, 56.9)	407 (326, 503)	7.2 (6.6, 7.9)	-85.4
Ardebil	3634 (3037, 4270)	102.6 (90.5, 115.6)	255 (211, 307)	12.6 (11.5, 13.7)	-87.7
Bushehr	1590 (1265, 1987)	62.5 (51.6, 75.4)	256 (204, 323)	12.1 (10.4, 14)	-80.6
Chahar Mahaal and Bakhtiari	1503 (1250, 1776)	60.8 (53.3, 68.7)	140 (111, 177)	7.8 (6.7, 9.1)	-87.1
East Azarbayejan	7844 (6511, 9314)	79 (69.4, 89.4)	587 (487, 704)	9.6 (8.8, 10.4)	-87.9
Fars	5949 (4929, 7033)	55.3 (48.4, 62.4)	927 (769, 1118)	12.4 (11.3, 13.4)	-77.6
Gilan	6504 (5701, 7436)	99.4 (88.6, 111.9)	162 (126, 205)	5.5 (4.5, 6.5)	-94.5
Golestan	3479 (2900, 4099)	77.2 (68.2, 87.1)	435 (363, 521)	12.4 (11.3, 13.4)	-84
Hamadan	4243 (3541, 5007)	85 (75, 95.8)	334 (277, 401)	13 (11.9, 14.1)	-84.7
Hormozgan	2781 (2320, 3265)	82.5 (72.6, 92.9)	476 (394, 572)	12.4 (11.4, 13.5)	-84.9
Ilam	1120 (938, 1315)	73 (64, 82.8)	108 (84, 138)	12.4 (10.2, 14.7)	-83.1
Isfahan	4841 (3882, 5985)	50.6 (42.5, 60.5)	673 (555, 809)	10.8 (9.9, 11.7)	-78.6
Kerman	3919 (3244, 4672)	66 (57.7, 75.1)	889 (741, 1069)	14.8 (13.6, 16)	-77.6
Kermanshah	4419 (3694, 5213)	84.7 (74.4, 95.7)	408 (338, 492)	13.1 (12, 14.2)	-84.6
Khorasan-e-Razavi	14847 (12260, 17482)	92.5 (80.8, 105.3)	1573 (1307, 1900)	12.2 (11.2, 13.2)	-86.8
Khuzestan	6306 (5296, 7481)	57.6 (50.7, 65.2)	1151 (960, 1384)	11.9 (10.9, 12.9)	-79.3
Kohgiluyeh and Boyer-Ahmad	1264 (1060, 1498)	67.3 (58.5, 76.5)	143 (112, 180)	9.4 (8.1, 11)	-86
Kurdistan	4535 (3823, 5314)	117.9 (103.6, 132.6)	351 (289, 424)	13 (11.9, 14.1)	-89
Lorestan	3512 (2937, 4130)	65.3 (56.7, 74.1)	216 (172, 273)	7.1 (6, 8.2)	-89.2
Markazi	2158 (1801, 2557)	68.5 (60, 77.7)	205 (162, 259)	10.8 (9.2, 12.4)	-84.3
Mazandaran	3143 (2540, 3805)	42.4 (35.8, 49.1)	322 (268, 383)	7.6 (6.9, 8.2)	-82.2
North Khorasan	2414 (2018, 2867)	96 (84.1, 109.2)	196 (155, 248)	12.2 (10.5, 14.2)	-87.2
Qazvin	1904 (1586, 2262)	74 (64.9, 83.8)	192 (152, 243)	9.5 (8.1, 11)	-87.2
Qom	1169 (951, 1415)	53.6 (45.6, 62)	190 (150, 240)	8.4 (7.2, 9.7)	-84.4
Semnan	758 (629, 899)	61.3 (54, 69.2)	111 (86, 142)	10.1 (8.4, 12)	-83.5
Sistan and Baluchistan	6122 (5126, 7173)	108.5 (95.2, 122.3)	1574 (1297, 1910)	17.9 (16.4, 19.4)	-83.5
South Khorasan	2464 (2052, 2912)	101.9 (90, 115.3)	211 (167, 267)	13.3 (11.4, 15.4)	-87
Tehran	7662 (6047, 9508)	32.1 (26.1, 38.7)	1501 (1239, 1811)	7.6 (6.9, 8.2)	-76.4
West Azarbayejan	6343 (5302, 7503)	85.7 (75.1, 97.2)	790 (655, 951)	12.3 (11.3, 13.3)	-85.7
Yazd	1040 (863, 1234)	50.8 (43.9, 58.5)	241 (191, 305)	12 (10.3, 13.9)	-76.4
Zanjan	7531 (6894, 8205)	81.1 (70.9, 91.7)	206 (163, 261)	12 (10.3, 13.9)	-85.2

###  Mortality by Cause

 Neonatal disorders, congenital birth defects, and lower respiratory infections ranked first to third among neonates, infants, and children, in both 1990 and 2019 (Figure 4). Among neonates, diarrheal diseases ranked 4th in 1990, while they ranked 6th in 2019 (Figure 4A). Additionally, among neonates in 1990, neonatal disorders accounted for 75.3% of all deaths, congenital birth defects accounted for 17.9% of all deaths, and lower respiratory infections accounted for 3.2% of deaths. Respective figures for 2019 were 70.6%, 23.8%, and 1.6%.

**Figure 4:**
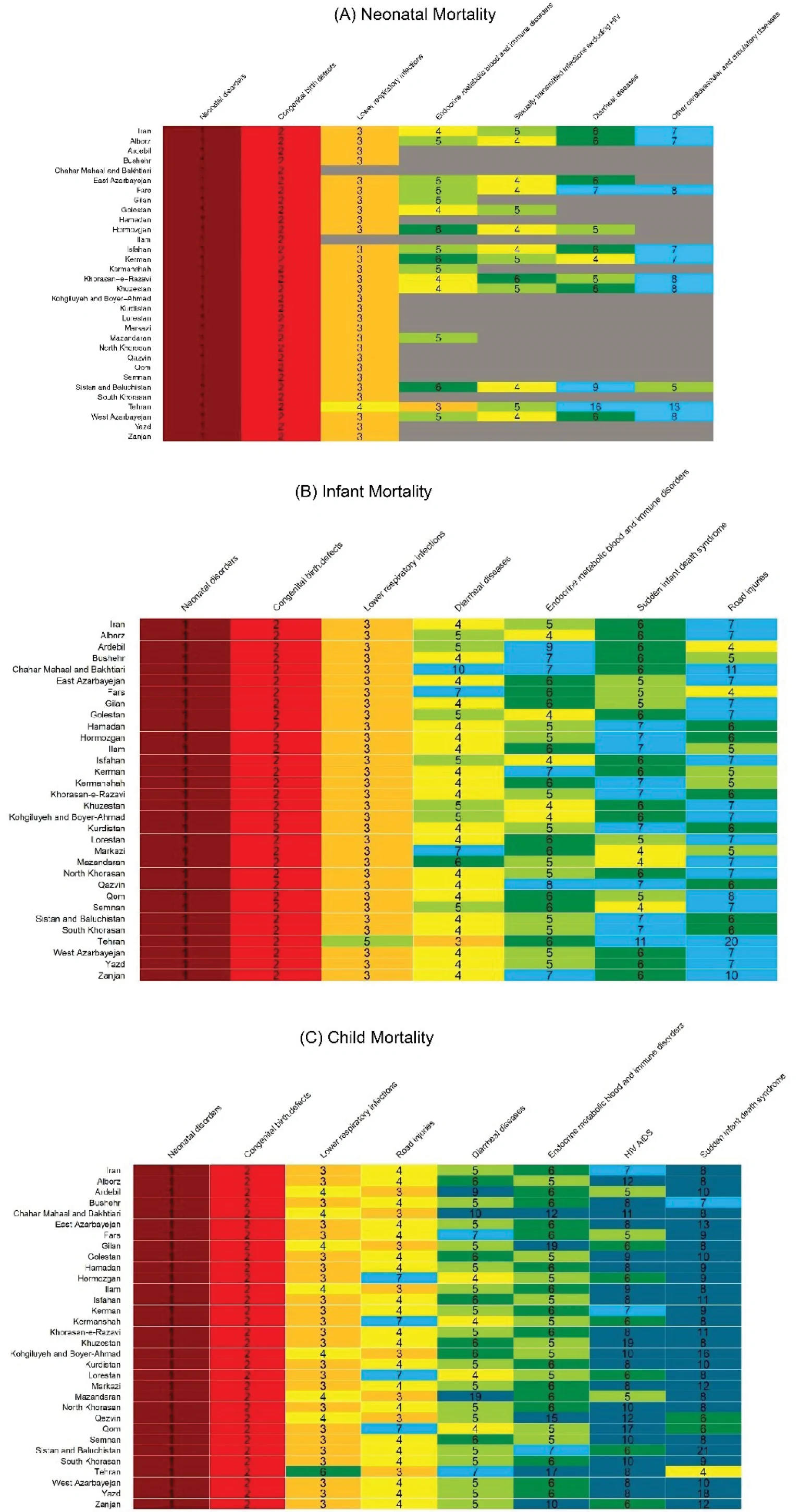
Neonatal, Infant, and Child Deaths by Cause in 2019. (**A)** Neonatal mortality.** (B) **Infant mortality. (**C)** Child mortality

 In 2019, the share of sub-causes of overall neonatal disorders among neonates were: neonatal preterm birth (62.7%), other neonatal disorders (22.0%), neonatal encephalopathy due to birth asphyxia and trauma (9.1%), neonatal sepsis and other neonatal infections (5.4%), and Hemolytic disease and other neonatal jaundice (0.9%). The rankings of neonatal disorders subtypes were the same in 1990.

 Neonatal disorders, congenital birth defects, and lower respiratory infections were the three main causes of infant and child mortality in almost all provinces of Iran. In infants, diarrheal diseases; endocrine, metabolic, blood, and immune disorders; sudden infant death syndrome, and road injuries were the next causes of death. The share of neonatal disorders out of all causes of infant mortality was 56.2% in 2019. Respective figures for congenital birth defects and lower respiratory infections were 28.8% and 3.5% in 2019 (Figure 4B).

 In children, however, road injuries; diarrheal diseases; endocrine, metabolic, blood, and immune disorders; HIV/AIDS; and sudden infant death syndrome were the next causes of mortality. The share of neonatal disorders out of the total causes of death among children under 5 years was 48.2%. Respective figures for congenital birth defects and lower respiratory infections were 27.6% and 3.8% in 2019 (Figure 4C).

Figure 5 demonstrates the provincial trends in neonatal, infant, and child mortality versus SDI from 1990 to 2019. Trends demonstrate an apparent decrease in mortality in all provinces and the convergence of trends, showing decreased inequality in the distribution of neonatal, infant, and child deaths in Iran since 1990.

**Figure 5:**
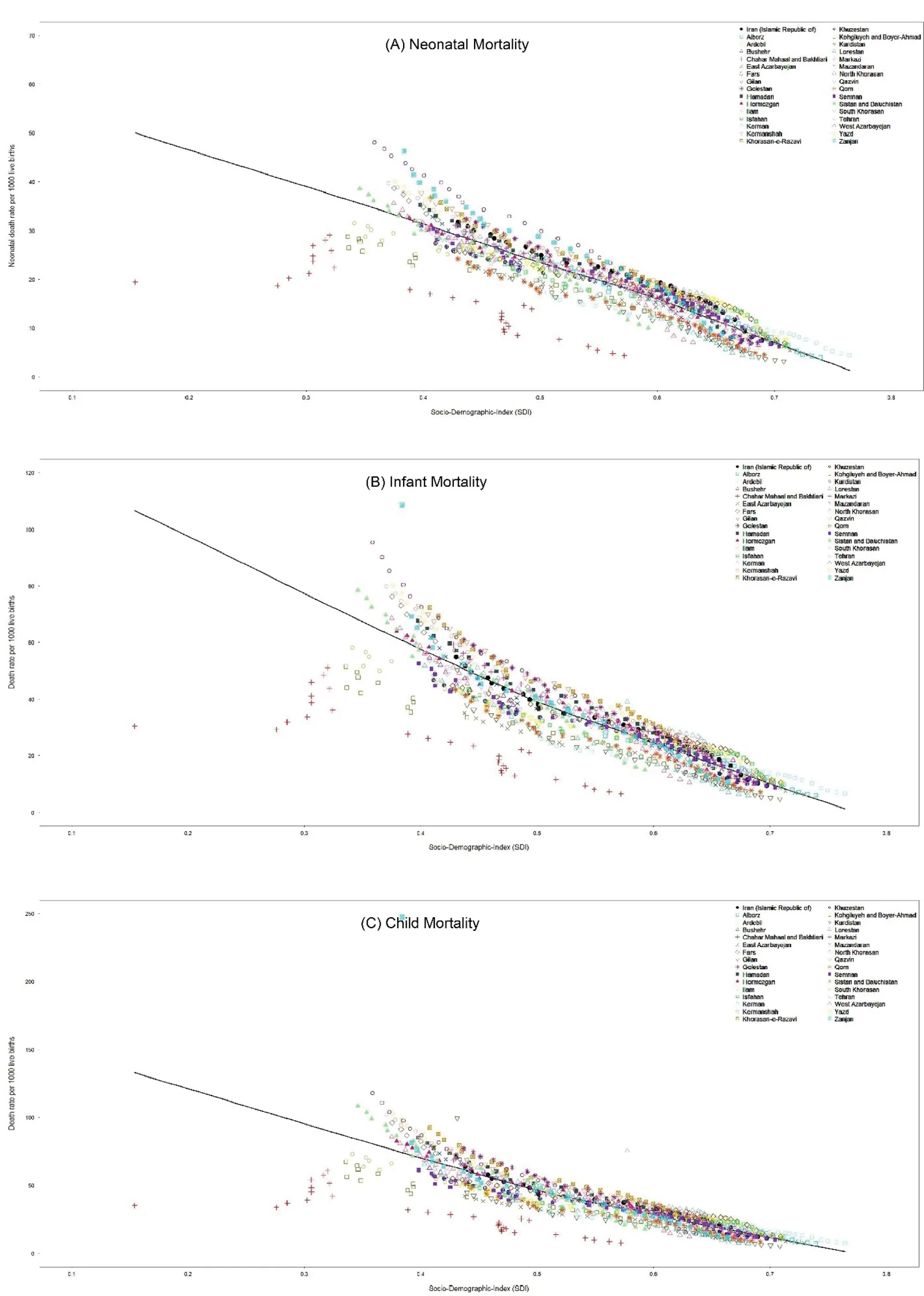
The Trend in Neonatal, Infant, and Child Mortality Along with Increase in Sociodemographic Index Across Provinces of Iran from 1990 to 2019. (**A)** Neonatal mortality. (**B)** Infant mortality. (**C)** Child mortality

## Discussion

 The results of our study demonstrated substantial decline in neonate, infant, and child mortality, counts and rates, in Iran from 1990 to 2019. The probability of dying within the first month and first year of life declined by 78.6% and 82.7% respectively during the past three decades. The probability of dying within the first five years of life also decreased by 84.4% during the same time period. All provinces of Iran achieved the SDG 3.2 target of neonatal mortality rate less than 12 per 1000 live births and child mortality rate less than 25 per 1000 live births in 2019. There was a convergence in trend of infant and child mortality along with increase in SDI from 1990 to 2019. Still, in 2019 the ratio of highest to lowest rates across provinces was 3.2 for both neonate and infant mortality, and 3.3 for child mortality, showing inequality in rates that are not negligible.

 UNICEF estimates for infant and child mortality rates were lower than our estimates for 1990 and higher for 2019. Based on UNICEF reports, Iran has achieved the SDG 3.2 target of child mortality less than 25 per 1000 live births.^3^ Estimates for child mortality by Mohammadi et al were also higher than our estimates for both 1990 and 2015, but they also confirmed that Iran has already achieved the SDG 3.2 target of child mortality less than 25 per 1000 live births. Mohammadi et al have chosen GBD methodology for their estimates and have considered both cross-sectional and over time uncertainties, making their estimates similar to ours.^19^

 The substantial decline in neonate, infant, and child mortality in Iran can be attributed to improvement in health and non-health infrastructure. The establishment of the primary health care network in 1980s in Iran can be considered as the first improvement in Iran’s health infrastructure that continued to the present.^26-28^ The expanded network consists of trained primary healthcare workers, who provide essential service packages for pregnant women and children free of charge in urban and particularly in rural areas.^29^ The service package includes integrated management of childhood health and child growth monitoring, child nutrition, immunization, promotion of breastfeeding, and family planning and pregnancy, all of which are crucial for child survival. Additionally, with the implementation of the National Maternal Mortality Surveillance System throughout Iran in 2001,^30^ maternal and neonatal mortality decreased substantially and the coverage of skilled birth attendance reached 100% in 2017, which paved the way towards universal health coverage in Iran.^5,31^ Later in 1990s and 2000s, with the integration of family physician program in the health system,^32,33^ the implementation of the health sector evolution plan,^34^ and the rural health system reform,^35^ the primary health care provision became more extensive and equitable.^36,37^ Expansion of access to hospital facilities and neonatal intensive care units, and access to specialized pediatric care are other improvements in health infrastructure of Iran that should not be neglected. Among non-health drivers of improvement in neonate, infant, and child mortality, women’s literacy, decreased child marriage, socio-economic development, access to safe drinking water and sanitation, and urbanization can be mentioned, which overall improved neonate, infant, and child health in Iran.^38^

 There are still studies that show disparities in neonate, infant, and child mortality across provinces of Iran.^12,13,16,18^ The results of the current study also showed that despite convergence of trends, there are still disparities in neonate, infant, and child mortality across provinces. Our results additionally demonstrated the reverse association between sociodemographic index and neonate, infant, and child mortality from 1990 to 2019. Disparities may be also due to uneven distribution of health care workers and hospital facilities; long distance to health centers; and local cultural and financial barriers to proper access to reproductive health care and family planning, contraceptive use, and prenatal, neonatal, intrapartum, and postnatal health care utilization, specifically in provinces with low SDI. Unconventionally young or old pregnancy age, multi-parity, and consanguine marriages are other potential determinants of high-risk pregnancies, leading to preterm births and congenital defects in low SDI provinces. Lack of universal routine vaccination for Haemophilus influenza type b and pneumococcal vaccines may be the cause of lower respiratory infections among neonates.

 To the best of our knowledge, the current study is the first that addresses neonate, infant, and child mortality by cause and by province in Iran during the past three decades. Yet, the current study has limitations similar to previous iterations of GBD, which mainly include lack of primary data. The incompleteness and misclassification of the vital registration data in Iran is one of the main limitations of this study. Another limitation of this study is the information bias in recorded data. The bias may be due to the fact that mothers may not exactly remember the date of birth and death of their children. The misclassification in the vital registration system of Iran may have had impacts on accuracy of cause of death estimations among infants and children. Finally, differences in the definition of live birth were an important limitation in this study.

 Future policy implications include implementing a unified guideline for child care across the entire country; providing high quality timely life-saving facilities for every mother and every neonate, infant, and child; coordinating policies to provide integrated services in different sectors; creating financial incentives to increase access to quality neonatal care services; establishment of a referral system for high-risk neonates; ensuring equal access to resources, essential medicines, and the necessary infrastructure for neonatal care in routine and emergency situations, and updating neonatal clinical care protocols to cover all levels of neonatal care. The ultimate aim of all mentioned policies should be the achievement of universal health coverage to fill the gaps in neonatal, infant, and child health between provinces in Iran. Policies should be focused on primary and secondary prenatal and neonatal care, as neonatal disorders and neonatal preterm births account for the majority of deaths among all neonates, infants, and children. Finally, policies should be tailored to specific geographical characteristics and local cultures in each province and should be aimed at raising the awareness and knowledge of parents towards healthy and safe motherhood.

 In conclusion, in this study we demonstrated a substantial improvement in neonate, infant, and child mortality in Iran during the past three decades and the fact that Iran has already achieved the SDG targets for neonate and child mortality rate. Still, there are considerable inequalities between provinces. With the probable continuation of sanctions on Iran, the continuing burden of COVID-19 pandemic, and the subsequent socioeconomic deterioration and food insecurity, decreased capacity of the health care system and attrition of health care workers are predictable. Future policies should be focused on maintaining the current infrastructure to provide the essential health care service to children and mothers and to set priorities and make policies based on evidence on burden of neonate and child mortality across provinces of Iran.
